# SMPDB 2.0: Big Improvements to the Small Molecule Pathway Database

**DOI:** 10.1093/nar/gkt1067

**Published:** 2013-11-06

**Authors:** Timothy Jewison, Yilu Su, Fatemeh Miri Disfany, Yongjie Liang, Craig Knox, Adam Maciejewski, Jenna Poelzer, Jessica Huynh, You Zhou, David Arndt, Yannick Djoumbou, Yifeng Liu, Lu Deng, An Chi Guo, Beomsoo Han, Allison Pon, Michael Wilson, Shahrzad Rafatnia, Philip Liu, David S. Wishart

**Affiliations:** ^1^Department of Computing Science, University of Alberta, Edmonton, AB, Canada T6G 2E8, ^2^Department of Biological Sciences, University of Alberta, Edmonton, AB, Canada T6G 2E8 and ^3^National Institute for Nanotechnology, 11421 Saskatchewan Drive, Edmonton, AB, Canada T6G 2M9

## Abstract

The Small Molecule Pathway Database (SMPDB, http://www.smpdb.ca) is a comprehensive, colorful, fully searchable and highly interactive database for visualizing human metabolic, drug action, drug metabolism, physiological activity and metabolic disease pathways. SMPDB contains >600 pathways with nearly 75% of its pathways not found in any other database. All SMPDB pathway diagrams are extensively hyperlinked and include detailed information on the relevant tissues, organs, organelles, subcellular compartments, protein cofactors, protein locations, metabolite locations, chemical structures and protein quaternary structures. Since its last release in 2010, SMPDB has undergone substantial upgrades and significant expansion. In particular, the total number of pathways in SMPDB has grown by >70%. Additionally, every previously entered pathway has been completely redrawn, standardized, corrected, updated and enhanced with additional molecular or cellular information. Many SMPDB pathways now include transporter proteins as well as much more physiological, tissue, target organ and reaction compartment data. Thanks to the development of a standardized pathway drawing tool (called PathWhiz) all SMPDB pathways are now much more easily drawn and far more rapidly updated. PathWhiz has also allowed all SMPDB pathways to be saved in a BioPAX format. Significant improvements to SMPDB’s visualization interface now make the browsing, selection, recoloring and zooming of pathways far easier and far more intuitive. Because of its utility and breadth of coverage, SMPDB is now integrated into several other databases including HMDB and DrugBank.

## INTRODUCTION

Biological pathways are the wiring diagrams of life. They provide a rich and surprisingly compact view of how genes, proteins and metabolites work together in cells, tissues or organs. In fact, most of today’s life scientists learned their biochemistry and molecular biology by studying richly annotated, carefully hand-drawn pathway diagrams found in books or on wall charts. With the advent of the internet, many biological pathway diagrams started migrating from the printed page to the web. As a result, there is now a plethora of very popular, very high-quality web-accessible pathway databases such as KEGG (1), the ‘Cyc’ databases (2,3), the Reactome database (4), WikiPathways (5) and PharmGKB (6). The advantages of using the web to illustrate and disseminate biological pathway information are manifold. These include greater accessibility, improved interactivity and enhanced functionality. However, the limitations associated with creating hyperlinked web illustrations often means that visual interactivity has to come at the expense of artistic quality or biochemical detail. This can be particularly problematic in the fields of clinical metabolomics and clinical chemistry where details about chemical structures as well as target organs, tissue locations, organelle function and physiological activity are particularly important.

In an effort to make web-based biological pathway data more visually appealing and physiologically relevant, particularly for biomedical applications, we developed the Small Molecule Pathway Database or SMPDB (7). The first version of SMPDB, which was released in 2010, contained >350, colorful, artistically rendered and biochemically complete pathway illustrations describing many key aspects of human metabolism. These included hyperlinked diagrams of human metabolic pathways, metabolic disease pathways, metabolite signaling pathways and drug-action pathways. Many of these pathway diagrams included information on the tissue locations, relevant organs, organelles, subcellular compartments, protein cofactors, protein locations, metabolite locations, chemical structures and protein quaternary structures. As with many high-quality web-based pathway databases, SMPDB also provided extensive hyperlinking, browsing, searching and annotation functions along with detailed pathway descriptions and references.

However, the first version of SMPDB was not without some shortcomings. In particular, it was difficult to update and maintain, it was limited in size and scope, its pathways lacked a certain degree of standardization, its physiological information on organelles and organs was incomplete and its visual interactivity (zooming and image navigation) was awkward and somewhat restricted. Furthermore, SMPDB pathway diagrams were only downloadable as static images and not available in standard formats such as BioPAX or SBML (8). While SMPDB did link to well-known databases such as DrugBank (9), HMDB (10) or UniProt (11), these databases did not link to it. In other words, SMPDB was not particularly visible to the metabolomics, drug research or systems biology communities.

With the release of SMPDB 2.0, we believe we have addressed all of these shortcomings. In particular, we have developed tools to simplify SMPDB’s maintenance and enhance its illustration standards. We have also corrected and added much more physiological (tissue, organ, organelle and transporter) information, substantially improved its visual interactivity and quality, created downloadable BioPAX pathway files for all of SMPDB’s pathways and increased SMPDB’s visibility by integrating SMPDB into DrugBank, HMDB and MetaboAnalyst. Finally, we have substantially expanded the size and scope of SMPDB to include >600 pathways (a 70% increase) and have added a large number of drug metabolism and physiological action pathways. With these enhancements, we believe that SMPDB 2.0 will appeal to a much broader community of researchers and will offer users a much more enriched, interactive and informative experience. A detailed description of SMPDB 2.0 follows.

## INCREASED SIZE AND SCOPE

When it was initially released, SMPDB (version 1.0) contained 350 hand-drawn pathways. These small molecule pathways were limited to three general categories: basic metabolism, metabolic diseases and selected drug-action pathways. Today, SMPDB 2.0 contains >600 pathways (a 70% increase) and it now includes six kinds of small molecule pathway categories including: basic metabolism, metabolic diseases, drug-action pathways, drug metabolism pathways, metabolite signaling pathways and physiological action pathways. The inclusion of drug metabolism pathways in SMPDB 2.0 was driven by user requests and the rapidly growing interest in drug metabolism in many fields of drug research and clinical metabolomics. The addition of metabolite signaling and physiological action pathways was motivated by the need to handle a larger number of drug-action pathways. It was also brought on by the growing awareness by members of the metabolomics and nutritional science communities that many metabolic pathways act at the level of organs and tissues and not only within cells. In addition to the significant numerical increase in SMPDB’s size and scope, many of the pathway diagrams in SMPDB 2.0 have been enhanced with additional cellular or physiological information. Now nearly every SMPDB pathway includes information about where the reactions occur (cellular compartment(s), organs or tissues), the site/organ of action for drugs (for drug-action pathways) or toxic metabolites (for metabolic disorders) as well as information on key membrane-bound transporters (which move drugs and metabolites in and out of almost every cell). This kind of physiological or extracellular information is rarely captured or displayed in other online pathway databases. However, because SMPDB is a human-only pathway resource, this sort of physiological, cellular, biochemical or biomedical information is particularly important. Consequently, considerable effort over the past 3 years has been put into capturing and displaying these data.

As with its predecessor, SMPDB 2.0 is still strictly focused on curating human-only, small molecule-only pathways. Therefore, SMPDB only displays those pathways where small molecules (metabolites or drugs <1500 daltons) play key roles and/or where small molecules represent a significant proportion of functional entities in a given pathway. All of the pathways in SMPDB 2.0 still retain the standard (and, in some cases, unique) database functionalities of the original database, including hyperlinked structures (to HMDB or DrugBank), hyperlinked protein images (to UniProt), text searching utilities (TextQuery), chemical searching utilities (ChemQuery), sequence searching utilities (Sequence Search), pathway browsing capabilities (SMP-Browse with filtering options), detailed pathway descriptions, extensive references, metabolite highlighting capabilities (SMP-Analyzer and SMP-Highlight), pathway legends and metabolite mapping for metabolomic applications (SMP-MAP). Additional details about these functions and how they can be used are provided in the original SMPDB paper. An updated and detailed comparison of SMPDB 2.0 to its predecessor (SMPDB 1.0) and to other pathway databases is provided in Table 1.

**Table 1.** Comparison of SMPDB 2.0 to SMPDB 1.0 to KEGG, HumanCyc, Reactome, BioCarta and WikiPathways/GenMAPP

Feature	SMPDB 2.0	SMPDB 1.0	KEGG	Reactome	HumanCyc	BioCarta	WikiPathways
Number of metabolic pathways	92	70	78 (for humans)	64 (for humans)	333 (short pathways)	69	50 (for humans)
Number of disease pathways	221	113	52	11	0	24	16
Number of drug action pathways	232	168	0	3	3	10	5
Number of drug metabolism pathways	53	0	8	1	0	0	2
Number of physio. action pathways	5	0	31	36	0	5	22
Number of small mol. signaling pathways	15	0	30	27	0	30	15
Provides multiple organism pathways	No	No	Yes	Yes	No	Yes	Yes
Chemical structures shown in diagrams	Yes	Yes	No	No	Yes (when zoomed)	Some	No
Protein 4^o^ structures shown in diagrams	Yes	Yes	No	No	No	Some	No
Cell structures shown in pathway diagrams	Yes	Yes	Some	Yes	No	Yes	No
Organs shown in pathway diagrams	Most	Some	No	No	No	No	No
Descriptions of pathways provided	Detailed	Detailed	Limited	Detailed	Detailed	Detailed	Limited
Pathway images are hyperlinked	Yes	Yes	Yes	Yes	Yes	Yes	No
Pathway images are easily zoomable	Yes	No	No	Yes	Yes	No	Limited
Information provided on pathway entities	Detailed	Detailed	Modest	Modest	Moderate	Limited	Limited
Supports advanced text search	Yes	Yes	No	Yes	Yes	No	No
Supports sequence searching	Yes	Yes	No	No	Yes	No	No
Supports graphical chem. structure search	Yes	Yes	No	No	No	No	No
Supports chemical expression mapping	Yes	Yes	Yes	Yes	Yes	No	No
Supports gene/protein expression mapping	Yes	Yes	Yes	Yes	Yes	No	No
Downloadable	Yes	Yes	Limited	Yes	Yes	No	Yes
BioPax, CellML or SBML compatible	Yes	No	Partial	Yes	Yes	No	No

## ENHANCEMENTS IN VISUALIZATION AND INTERACTIVITY

One of the distinguishing features of SMPDB has been the strong focus on providing users with high-quality, artistically pleasing pathway diagrams that were not only correct and informative but also colorful, interactive and richly detailed. This continues to be a major focus for SMPDB 2.0. To this end, a significant effort over the past year has been put into enhancing the visual displays and interactivity in SMPDB. The previous version of SMPDB only offered a three-step zooming capability that was further hindered by the requirement that users navigate through the zoomed-in images using multiple scroll bars. This restricted zooming capability proved to be both awkward and inconvenient for many users. Likewise, the placement of the pathway descriptions and references (above and below the pathway images) limited how much of a pathway could be viewed on a web page or computer screen. In addition, the size of the chemical structures (too large), the text labels (too small) and the pathway arrows (too thin) was often problematic for a number of pathways, depending on what level of zooming was used. The uniformly dark blue background (to indicate an aqueous environment) was also challenging for those wishing to print SMPDB pathways on paper, for preparing slides and for visualizing certain features or distinguishing among subcellular locations.

**Figure 1.**
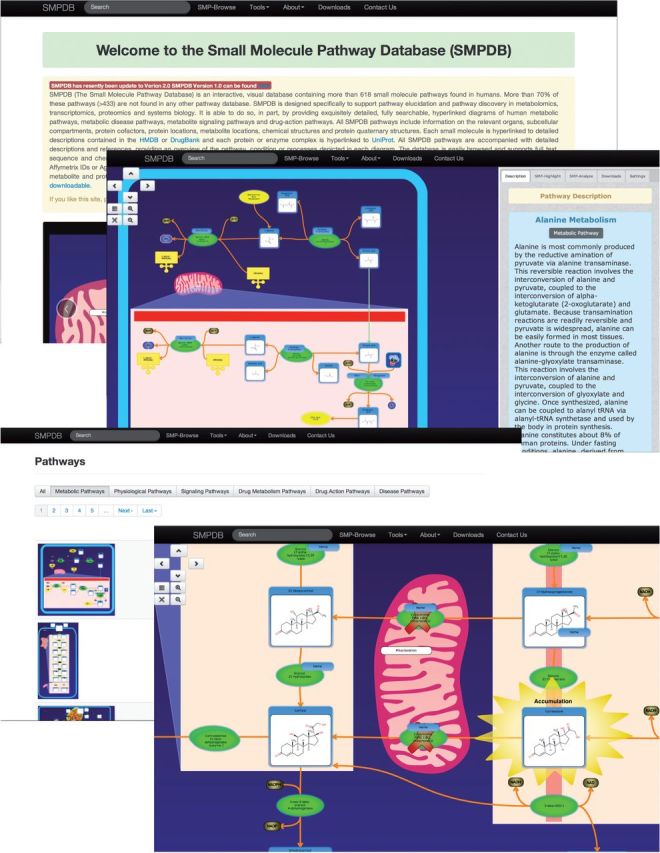
A screenshot montage of SMPDB 2.0’s various viewing and searching features.

For SMPDB 2.0, a completely new visualization engine was built using scalable vector graphics (SVG) and a web interface technology inspired by Google Maps (http://en.wikipedia.org/wiki/Google_Maps). This enhancement now allows rapid and continuous zooming using a mouse scroll wheel or through simply clicking on-screen zoom icons. It also allows facile navigation around zoomed-in pathway diagrams in SMPDB 2.0 through a simple click-and-drag operation or through clicking on-screen up/down or left/right arrows located near the on-screen zoom functions. A full-screen view of each SMPDB map is also available. This view can be toggled off and on by clicking the full-screen icon located on SMPDB’s new information and control panel (located on the right side of each pathway map). The information and control panel allows users to easily click on labeled buttons to view text (pathway descriptions and references), highlight (SMP-Highlight), annotate concentration data (SMP-Analyze), download files and adjust image settings. By moving the old text boxes containing pathway descriptions and pathway references to SMPDB 2.0’s information and control panel, more on-screen real estate is now available for viewing pathway diagrams. Furthermore, by resizing, recoloring and standardizing the size of the chemical structures, arrows and text in all of SMPDB’s pathway images, most features under most zooming conditions should be much more visible and easily discerned. Using the image settings located in the control panel, users can also adjust the background colors (from blue to white) and the cellular membrane display (simple versus detailed) to facilitate printing, image capture or slide preparation. The recoloring process has also been extended to other parts of SMPDB as well. In particular, all reactions that take place in certain organelles or subcellular locations have now been recolored so that the organelle’s background color serves as the background color for the (zoomed-in) reaction. This should make compartment-specific reactions or pathways far more distinctive and discernable. Another enhancement to SMPDB is now available through the home page where a scrollable ‘carousel’ of SMPDB pathway images is available. This carousel, which can be scrolled through by simply ‘swiping’ the mouse over the images, will allow users to view and select newly loaded or recently updated SMPDB pathway maps. A montage of many of these visualization features and enhancements is shown in Figure 1.

## IMPROVED STANDARDIZATION AND REDUCED MAINTENANCE

SMPDB was originally built by hand-drawing each pathway using PowerPoint incorporating a collection of visual icons and a well-defined standard operating protocol (SOP). Each PowerPoint image was then converted to three differently sized PNG images (small, medium and large) and manually image-mapped to appropriate database links. Prior to the image mapping process, pathway constituents (proteins and chemicals) were manually identified, their database IDs manually determined and each data point entered in a separate file for each SMPDB pathway. These text files were used to permit text and structure searching as well as compound highlighting (via SMP-MAP and SMP-Highlight). This manually intensive process proved to be arduous, time-consuming and error prone. The many different skills and multiple steps required to generate and post a SMPDB pathway meant that a team of more than 10 differently trained individuals were needed to construct, update and maintain the database. Even with SOPs and a standardized icon library, some individual variability occurred with regard to pathway layout, pathway details, icon placement, directional arrow size and other image features. Additionally, the manual placement of image-mapped hyperlinks meant that some links were placed imprecisely, leading to off-centre effects that became further exacerbated through zooming. If SMPDB pathways needed to be updated or corrected, multiple images still had to be generated and multiple image maps still had to be prepared. If a large number of highly similar pathways were involved (such as drug mechanism pathways) a simple, single molecule update could often take many hours of repetitive, error-prone editing. Because all of SMPDB’s pathways were essentially image-mapped pictures it was not possible to easily render them into BioPAX or SBML formats. As a result, efforts to convert SMPDB pathways into BioPAX or SBML equivalents were stymied.

To simplify the updating process and improve the level of standardization and figure rendering consistency for SMPDB 2.0, we developed an online pathway illustration and editing tool, called PathWhiz (manuscript in preparation). PathWhiz, which is a combination of an online digital painting program and a webpage building program, allowed SMPDB curators to digitally render, update and annotate pathways in a fraction of the time that the old ‘analog’ approach required. PathWhiz also allowed our curation team to produce far more consistently rendered pathways and consistently sized structures that looked as if they all came from a single artist. PathWhiz uses a pull-down library of standard image icons for membranes, membrane compartments, organs, tissues, organelles, membranes and proteins to help accelerate the pathway illustration process. It also has a standard set of arrows and arrow-drawing utilities that allow all SMPDB reaction components to be linked or connected in a uniform, visually pleasing fashion. Because PathWhiz also captures icon placement and annotation information digitally, it allowed all hyperlinks to be precisely mapped, all pathway images to be rendered as SVG and all pathway component information (names, structures, database IDs, etc.) to be readily captured. The updating process is also made much easier as all changes in one pathway (images, hyperlinks, names, database IDs) can be digitally transferred to all other similar pathways at the click of a button. The digital nature of the drawing and icon placement process through PathWhiz meant that it also became very easy to convert all SMPDB 2.0 pathways into BioPAX format. Conversion of the SMPDB pathways to SBML is underway now and should be completed before 2014. Thanks to PathWhiz, all SMPDB pathways have been significantly updated, corrected and improved. Likewise many new pathways were able to be added in a fraction of the time (compared to the old approach), allowing a significant increase in both the quantity and quality of pathways in SMPDB 2.0.

## ENHANCED DATA DOWNLOADS

Another important, and increasingly unique, feature of SMPDB is the open availability of its data. In particular, SMPDB supports both data downloads and image downloads for all of its pathways. The data files include protein and gene sequences for all the annotated proteins and genes in SMPDB. SMPDB downloads also include all metabolite and drug structures (in SDF format) as well as CSV files containing different combinations of compound names or protein names linked to pathways and database identifiers. The image files (in SMPDB 1.0) included both the original PowerPoint files and the differently sized (small, medium, large) PNG files corresponding to each pathway. With the release of SMPDB 2.0 the same kind of data download information is still available, although the number of sequences, structures and database links is now substantially greater. In addition, SMPDB’s data downloads now include all of its pathway information in BioPAX format. BioPAX is an RDF/OWL-based standard exchange language designed to compactly represent biological pathways at the molecular and cellular level (12). The availability of SMPDB’s data in BioPAX format and the impending availability of the same pathway data in SBML (expected in late 2013) should greatly expand SMPDB’s appeal to systems biologists as well as its potential utility in a variety of pathway, biochemical and metabolomic analysis packages. It is also expected that the availability of SMPDB BioPAX (and SBML) files will encourage greater interest in community annotation, allowing SMPDB to evolve from a centrally curated database (which is inefficient) to one that may be curated and enhanced by a community of experts (which is more efficient). SMPDB 2.0’s image file downloads have also been modified and enhanced to include not only high-resolution PNG files but also a SVG images of every pathway with all of the BioPAX data imbedded (as metadata) into every file. With these enhancements to SMPDB 2.0’s downloadable resources, we believe it now offers the most comprehensive collection of downloadable data of any online pathway database.

## IMPROVED QUALITY AND QUALITY ASSURANCE

As noted earlier, every pathway in SMPDB 2.0 has been redrawn using the new PathWhiz rendering tool. This effort allowed us to revisit all of SMPDB’s pathways and to update them with new information and to correct, improve or reformat preexisting pathways so that they were more consistent and informative. Many pathways were improved through the addition of more organelle and reaction compartment information. Others were enhanced through the addition of target organ information and protein transporter information. Likewise images that were too small, arrows that were too thin and pathways that were too convoluted were corrected and/or simplified. In redrawing old pathways and rendering new pathways in SMPDB 2.0, the same level of quality assurance, the same referential resources and the same kinds of data checking/validation routines were used as described in the original SMPDB paper (7). In particular, all pathways in SMPDB 2.0 were drawn using a standard operating procedure (SOP) with a checklist of features that each of the pathway artists/annotators was required to follow. This SOP and checklist is also provided in the ‘About’ menu. To ensure that the pathways have been knowledgably illustrated, all of the SMPDB pathway curators were required to have degrees or advanced university coursework in biology, chemistry, biochemistry or bioinformatics. During the redrawing of existing SMPDB pathways and the generation of new pathways for SMPDB 2.0, all SMPDB curators were also required to consult a variety of sources including biochemistry textbooks, OMIM (13), Wikipedia, KEGG (1), MetaCyc (3), Reactome (4), UniProt (11), the HMDB (10), DrugBank (9) and PharmGKB (6). This allowed the curation team to identify and consolidate pathway nomenclature, key pathway components, critical reactions, cellular compartments as well as target organs, compartments or organelles. Pathway layouts were also frequently assessed, compared and discussed by SMPDB team members prior to manually generating any pathway diagrams. Because of the unique rendering requirements and the strict SOPs for SMPDB, no pathway in SMPDB was ‘copied’ from any other pathway diagram in any other database. Furthermore, SMPDB’s metabolic disease pathways, drug-action and drug metabolism pathways had to be generated independently of any pathway database. Instead, relevant information was gathered from various medical and pharmacology textbooks, specialized encyclopedias and fragmentary data contained on several online databases such as OMIM (13), PharmGKB (6) and DrugBank (9). As an additional layer of quality assurance, all of the pathway diagrams in the SMPDB 2.0 have been inspected and corrected by two or more curators having advanced degrees in biochemistry or physiology.

## INCREASED CONNECTIVITY AND INTEROPERABILITY

When SMPDB was first released, it was primarily a stand-alone database that simply accessed other databases through its hyperlinks to HMDB, DrugBank and UniProt. Over the past 2 years, efforts have been directed at integrating SMPDB into a number of other popular online databases, so that effectively other databases now access SMPDB. In particular, SMPDB is now linked into the pathway data fields of the latest version of HMDB (10) and the latest release of DrugBank (9). It has also been linked into several online metabolomic data analysis resources including MetaboAnalyst (14) and MSEA (15). Discussions are ongoing to have SMPDB 2.0 linked to a number of other chemical entity databases as well as other well-known pathway databases in the near future. In addition to trying to increase SMPDB’s database connectivity, we are also trying to make it far more interoperable. The availability of SMPDB’s data in BioPAX format and the expected availability of the same pathway data in SBML (in late 2013) should greatly improve SMPDB’s interoperability. It should also encourage users to freely exchange SMPDB data and to incorporate these data into a variety of pathway or metabolomic analysis software packages. It is also expected that the availability of SMPDB BioPAX (and soon SBML) files will encourage greater interest in community annotation, allowing SMPDB to evolve from a centrally curated database to a community curated resource.

## FUTURE PLANS AND CONCLUSIONS

SMPDB continues to expand with new pathways being added at an almost daily rate. Over the coming 2–3 years, we expect that the number of pathways in SMPDB will likely double. It is likely that the greatest growth in SMPDB’s pathways will be in the drug and drug metabolism pathways, as there are literally thousands of reactions, reactants and pathways that are readily available in the literature. With the impending release of PathWhiz (SMPDB’s pathway drawing tool and editor), we are hopeful that many of SMPDB’s new pathways will actually be added by interested community members. It is also expected that PathWhiz will enable SMPDB-like pathways and pathway databases to be easily generated for other model organisms such as *Saccharomyces cerevisae*, *Escherichia coli*, Arabidopsis and Drosophila. With the latest additions and enhancements in SMPDB 2.0, we believe that SMPDB has reached a critical threshold giving it sufficient breadth, depth and interconnectivity so that it will appeal to a much larger community of users. Overall, SMPDB 2.0’s new, colorful, informative, artfully designed pathway diagrams, combined with its wide range of visualization, annotation and querying tools should provide users a much more enriched, interactive and informative pathway viewing experience.

## FUNDING

Canadian Institutes for Health Research (CIHR), Alberta Innovates Health Solutions (AIHS) and Genome Alberta, a division of Genome Canada. Funding for open access charge: Genome Canada.

*Conflict of interest statement.* None declared.
